# Supplementary material for: Metagenomic binning with assembly graph embeddings
Source: Bioinformatics. 2022 Aug 16;38(19):4481–7. doi: 10.1093/bioinformatics/btac557 (PMC9525014; doi:10.1093/bioinformatics/btac557)
Supplement: btac557_supplementary_data [file btac557_supplementary_data.zip › amber outputs.zip!/amber outputs/simHC/genome/vamb_edges/completeness_vs_genome_size_simHC+.pdf]

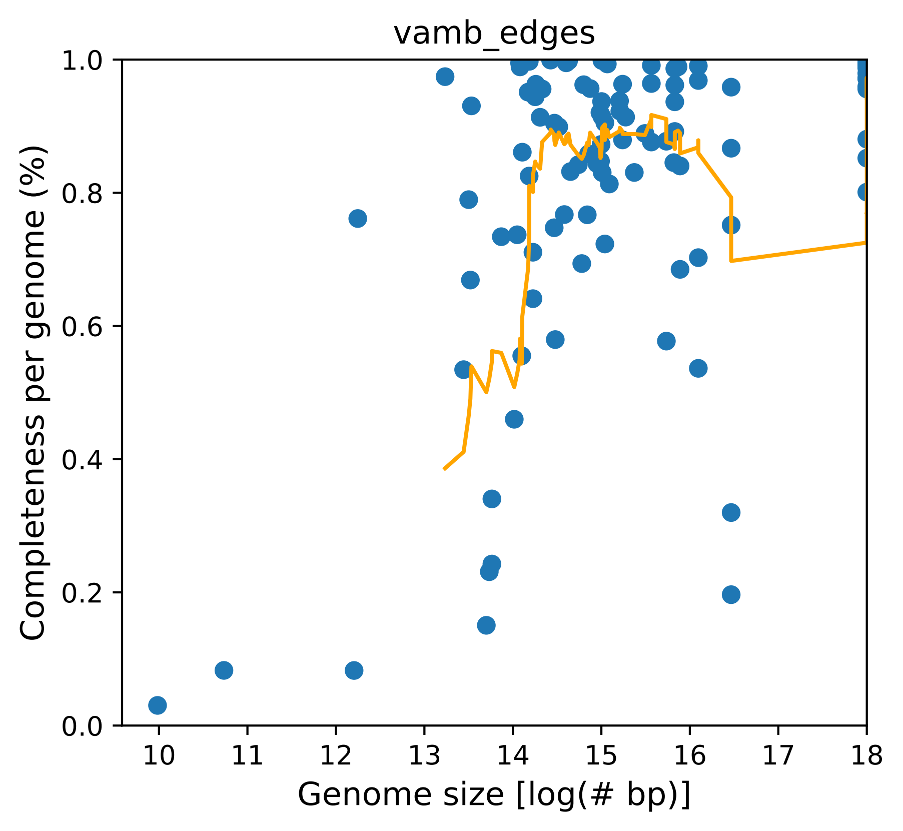

vamb_edges
Completeness per genome (%)
Genome size [log(# bp)]
0.0
0.2
0.4
0.6
0.8
1.0
10
11
12
13
14
15
16
17
18
